# 1,25(OH)_2_D_3_ Differently Affects Immunomodulatory Activities of Mesenchymal Stem Cells Depending on the Presence of TNF-α, IL-1β and IFN-γ

**DOI:** 10.3390/jcm8122211

**Published:** 2019-12-14

**Authors:** Christian Behm, Alice Blufstein, Johannes Gahn, Barbara Kubin, Michael Nemec, Andreas Moritz, Xiaohui Rausch-Fan, Oleh Andrukhov

**Affiliations:** 1 Department of Conservative Dentistry and Periodontology, University Clinic of Dentistry, Medical University of Vienna, 1090 Wien, Austria; christian.behm@meduniwien.ac.at (C.B.); alice.blufstein@meduniwien.ac.at (A.B.); johannes.gahn@gmail.com (J.G.); barbara.kubin@kabsi.com (B.K.); andreas.moritz@meduniwien.ac.at (A.M.); xiaohui.rausch-fan@meduniwien.ac.at (X.R.-F.); 2 Clinical Division of Orthodontics, University Clinic of Dentistry, Medical University of Vienna, 1090 Wien, Austria; michael.nemec@meduniwien.ac.at

**Keywords:** periodontal ligament, mesenchymal stem cells, CD4-positive T-lymphocytes, macrophages, calcitriol, cytokines, immunomodulation

## Abstract

Periodontal ligament-derived mesenchymal stem cells (hPDLSCs) possess immunomodulatory abilities which are strongly enhanced by various inflammatory cytokines. Vitamin D_3_ has anti-inflammatory effects on hPDLSCs and immune cells. However, no study to date has directly compared the influence of 1,25(OH)_2_D_3_ on the immunomodulatory activities of hPDLSCs in the presence of different cytokines. In the present study, the effects of hPDLSCs treated with tumor necrosis factor (TNF)-α, interleukin (IL)-1β, or interferon (IFN)-γ in the presence of 1,25(OH)_2_D_3_ on the proliferation of allogenic CD4^+^ T lymphocyte or on the functional status of primary CD68^+^ macrophages were analyzed in coculture models. Additionally, the effects of 1,25(OH)_2_D_3_ on TNF-α-, IL-1β-, and IFN-γ-induced gene expression of some immunomodulatory factors in hPDLSCs were compared. Under coculture conditions, 1,25(OH)_2_D_3_ increased or decreased CD4^+^ T lymphocyte proliferation via hPDLSCs, depending on the cytokine. hPDLSCs primed with 1,25(OH)_2_D_3_ and different cytokines affected pro- and anti-inflammatory cytokine expression in macrophages variably, depending on the priming cytokine. With one exception, 1,25(OH)_2_D_3_ significantly reduced TNF-α-, IL-1β-, and IFN-γ-induced expression of all the investigated immunomediators in hPDLSCs, albeit to different extents. These results suggest that 1,25(OH)_2_D_3_ influences the immunomodulatory activities of hPDLSCs depending qualitatively and quantitatively on the presence of certain inflammatory cytokines.

## 1. Introduction

Vitamin D_3_ is a hormone involved in controlling bone and calcium homeostasis [1] and in regulating immune response [2]. It is synthesized from 7-dehydrocholesterol by exposure to sunlight by the skin, or is obtained from dietary supplements. In the liver, previtamin D_3_ is further metabolized to 25(OH)D_3_, and finally by kidney to the biologically-active 1,25(OH)_2_D_3_ [1,3]. Local extrarenal conversion of 25(OH)D_3_ to 1,25(OH)_2_D_3_ occurs in different cells, particularly in immune cells [4,5] and periodontal ligament-derived mesenchymal stem cells (hPDLSCs) [6,7].

The periodontal ligament is a connective tissue linking the roots of teeth to the alveolar bone [8]. In 2004, it was described as a source for mesenchymal stem cells (MSCs) for the first time [9]. hPDLSCs are a heterogeneous cell population, showing a fibroblast-like morphology [10]. They are multipotent and non-hematopoietic progenitor cells having a self-renewal potential [11]. They fulfil the minimal criteria for MSCs, i.e., (1) expression of specific surface markers, and (2) the potential of osteogenic, adipogenic, and chondrogenic differentiation in vitro [12]. Within the periodontal ligament, they reside in the perivascular area in a quiescent and undifferentiated status [13,14]. Local inflammatory processes trigger hPDLSC migration to injured and inflamed periodontal tissue, mainly through chemotaxis. There, they are involved in controlling periodontal tissue regeneration and homeostasis, mainly by influencing local immune responses [15,16,17].

Like MSCs from other tissues, hPDLSCs mainly influence various immune cells such as by suppressing T lymphocyte proliferation or driving macrophages into a more anti-inflammatory functional status [18,19]. hPDLSCs fulfil these immunomodulatory activities by producing numerous enzymes and soluble factors such as indoleamine-2,3-dioxygenase-1 (IDO-1), prostaglandin E_2_ (PGE_2_), and tumor necrosis factor-inducible gene 6 protein (TSG-6) [19]. Immunomodulatory activities are usually low in resting hPDLSCs, and are boosted by environmental stimuli, such as inflammatory cytokines like tumor necrosis factor-α (TNF-α), interleukin-1β (IL-1β), and interferon-γ (IFN-γ), which are secreted by immune cells [20,21,22,23]. This implies a tight reciprocal interaction between hPDLSCs and immune cells [18], whose exact physiological role needs to be clarified.

The anti-inflammatory properties of vitamin D_3_ have been well recognized by numerous in vitro studies using different cell types [24,25], and particularly, hPDLSCs [7]. However, the data of clinical studies on the role of vitamin D_3_ in inflammatory diseases remain inconsistent. On the one hand, several inflammatory diseases and their severity are associated with vitamin D_3_ deficiency [26,27]. On the other hand, dietary supplementation of vitamin D_3_ in different diseases often has no positive systemic anti-inflammatory effect [28,29]. Regarding periodontal disease, the data on the role of vitamin D_3_ are incomplete. On the one hand, the majority of studies with few exceptions suggest an association of vitamin D_3_ deficiency with incidence and severity of periodontitis [30,31,32]. On the other hand, to the best of our knowledge, there is no evidence of the beneficial effects of vitamin D_3_ supplementation during the initial nonsurgical treatment phase. The mechanisms underlying the existing inconsistency between in vitro and clinical data regarding the anti-inflammatory role of vitamin D_3_ are complex [29]. In in vivo situations, vitamin D_3_ influences simultaneously not only several cell types, but also the interactions between them. For example, several studies have already shown that 1,25(OH)_2_D_3_ suppresses the production of pro-inflammatory cytokines in hPDLSCs [7,33,34,35]. These findings imply that vitamin D_3_ will also influence the interaction between hPDLSCs and immune cells, but this question remains to be explored.

Hence, the main aim of this study was to directly compare the influence of 1,25(OH)_2_D_3_ on the immunomodulatory activities of hPDLSCs. We investigated the effects of vitamin D_3_ in the presence of either TNF-α, IL-1β, or IFN-γ, because these cytokines activate different signaling pathways, and thus, different immunomodulatory aspects. In this in vitro study, we focused on the hPDLSC effect on the proliferation of CD4^+^ T lymphocytes and on the functional status of human primary macrophages. Further, the effect of 1,25(OH)_2_D_3_ on TNF-α-, IL-1β-, and IFN-γ-induced expression of IDO-1, prostaglandin-endoperoxide synthase (PTGS)-2, and TSG-6 in hPDLSCs was directly compared. The results indicate that 1,25(OH)_2_D_3_ modulates the immunomodulatory activities of hPDLSCs against CD4^+^ T lymphocytes and CD68^+^ macrophages differently, depending on the inflammatory cytokine context.

## 2. Experimental Section

### 2.1. Ethics

The isolation of primary hPDLSCs from patients and CD4^+^ T lymphocyte and CD14^+^ monocyte isolation from whole blood, as well as the study protocol, were approved by the Ethics Committee of the Medical University of Vienna (EK-Nr. 1694/2015, extended in October 2019). All experimental procedures were performed according to the Declaration of Helsinki and the Good Scientific Practice Guidelines of the Medical University of Vienna.

### 2.2. Primary hPDLSC Isolation and Cultivation

Third molars were extracted from five periodontally-healthy patients aged between 18 to 30 years due to orthodontic reasons. Before surgical extraction, the patients were informed and gave their written informed consent. Primary hPDLSCs were isolated from the periodontal ligament of the mid-third surface of the extracted tooth’s root, as described in our previous study [36]. They were cultivated in Dulbecco’s modified Eagles Medium (DMEM, Sigma-Aldrich, St. Louis, MO, USA), supplemented with 10% fetal bovine serum (FBS, Gibco, Carlsbad, CA, USA) and 1% penicillin and streptomycin (P/S, Gibco, Carlsbad, CA, USA) under humidified conditions. The stemness of isolated hPDLSCs was verified according to our recent study determining the positive expression of characteristic mesenchymal (CD29, CD44, CD73, CD90, CD105, and CD146) and negative expression of hematopoietic (CD14, CD31, CD34, and CD45) stem cell markers [37]. Primary hPDLSCs from passage five to seven were used for experiments.

### 2.3. Isolation of Primary CD4^+^ T Lymphocytes and CD14^+^ Monocytes

Heparinized whole blood was collected from volunteers using a lithium- and heparin-containing VACUETTE^®^ blood collection system (Greiner Bio-one, Kremsmünster, Austria). To reduce donor variability and to concentrate exclusively on the effect of hPDLSCs from different individuals, blood was collected from one single volunteer for CD14^+^ monocyte and from another single volunteer for CD4^+^ T lymphocyte isolation, respectively. Blood was diluted 1:1 with Hank’s Balanced Salt Solution (HBSS, Life Technology, Carlsbad, CA, USA), followed by Ficoll density gradient centrifugation (GE Healthcare, Chicago, IL, USA) to isolate allogenic peripheral blood mononuclear cells (PBMCs). PBMCs were used to isolate CD4^+^ T lymphocytes and CD14^+^ monocytes using MagniSort^TM^ Human CD4^+^ T cell enrichment kit and MagniSort^TM^ Human CD14 positive isolation kit (Invitrogen, Carlsbad, CA, USA), respectively.

### 2.4. CD4^+^ T Lymphocyte Proliferation in Coculture with hPDLSCs

First, 2.5 × 10^5^ primary hPDLSCs were seeded per well in 6-well plates using 3 mL DMEM, supplemented with 10% FBS and 1% P/S. After 24 h, the medium was replaced with serum-free DMEM to avoid an effect of FBS on the hPDLSC expression of immunomediators. hPDLSCs were treated with either 10 ng/mL TNF-α or 5 ng/mL IL-1β or 100 ng/mL IFN-γ (all from PeproTech, Rocky Hill, NJ, USA) in the absence or presence of 100 nM 1,25(OH)_2_D_3_ (Cayman Chemical, Ann Arbor, MI, USA) for 48 h. Afterwards, the medium was changed to Roswell Park Memorial Institute (RPMI) -1640 medium (Sigma-Aldrich, St. Louis, MO, USA), supplemented with 10% FBS and 1% P/S and corresponding stimuli. The RPMI-1640 medium was chosen because it provides optimal conditions for CD4^+^ T lymphocyte proliferation. Transwell (TC) inserts (0.4 µM pore size, Sarstedt, Nürnbrecht, Germany) containing 1 × 10^6^ allogenic CD4^+^ T lymphocytes in 1ml RPMI-1640 medium were placed into wells with prestimulated hPDLSCs. Before seeding, CD4^+^ T lymphocytes were labeled using the CellTrace CFSE Cell Proliferation Kit (ThermoFischer Scientific, Waltham, MA, USA), according to the manufacturer’s protocol. CD4^+^ T lymphocyte proliferation was activated by adding 10 µg/mL phytohemagglutinin (PHA-L, eBioscience, San Diego, CA, USA). After five days of additional incubation, CD4^+^ T lymphocytes were harvested and resuspended in a 3% bovine serum albumin solution (BSA, GE Healthcare, Chicago, IL, USA), supplemented with 0.09% sodium azid (Merck, Darmstadt, Germany). CD4^+^ T lymphocyte proliferation was determined using FACSCalibur Flow Cytometer (Becton Dickinson, Franklin Lakes, NJ, USA). An argon laser was used to excite fluorescence at 488 nm for a total of 10,000 cells. All cells were taken for analysis, and no additional gating was performed. The percentage of at least once-divided CD4^+^ T lymphocytes was determined using CellQuest 3.3 software (Becton Dickinson, Franklin Lakes, NJ, USA). PHA-L activated CD4^+^ T lymphocytes cultivated in the absence of hPDLSCs with the aforementioned cytokines and 1,25(OH)_2_D_3_ functioned as a control.

### 2.5. hPDLSCs and Macrophage Indirect Coculture

Next, 1.25 × 10^6^ allogenic CD14^+^ monocytes were seeded per well in 6-well plates using 1 mL RPMI-1640 medium supplemented with 10% FBS and 1% P/S. Macrophage differentiation was initiated by adding 50 ng/mL macrophage colony-stimulating factor (M-CSF, PeproTech, Rocky Hill, NJ, USA) for seven days. Macrophage differentiation and purity were confirmed by staining with anti-CD68 antibody (see Figure S1). In parallel, four days after starting macrophage differentiation, primary hPDLSCs were seeded in TC-inserts (0.4 µM pore size, Sarstedt, Nürnbrecht, Germany) at a density of 1.25 × 10^5^ cells per insert in 1ml complete DMEM medium. After 24 h, hPDLSCs were primed with either 10 ng/mL TNF-α or 5 ng/mL IL-1β or 100 ng/mL IFN-γ in the absence and presence of 100 nM 1,25(OH)_2_D_3_ for an additional 48 h. TC-inserts with primed hPDLSCs were applied to in vitro differentiated macrophages. Coculture was rinsed twice with 1× PBS, and the medium was replaced by RPMI-1640 supplemented with 2% FBS and 1% P/S. RPMI-1640 medium was utilized to ensure optimal conditions for macrophages in vitro. After an additional 24 h, the functional status of macrophages was verified by quantitative polymerase chain reaction (qPCR) analysis. The coculture of macrophages with unprimed hPDLSCs served as control.

The macrophage total RNA was isolated using RNeasy Mini Kit (Qiagen, Hilden, Germany) according to the manufacturer’s instructions. Extracted RNA was reverse transcribed into cDNA using High-Capacity cDNA Reverse Transcription Kit (ThermoFischer Scientific, Waltham, MA, USA). Samples were heated using Primus 96 advanced thermocycler (PeqLab/VWR, Darmstadt, Germany) to 25 °C for 10 min, to 37 °C for 120 min, and finally, to 85 °C for five minutes. For qPCR TaqMan Gene Expression Cells-to-CT Kit (Applied Biosystems, Foster City, CA, USA) and QuantStudio 3 device (Applied Biosystems, Foster City, CA, USA) were used. Samples were heated to 95 °C for 10 min, which was followed by 50 cycles of 15 s at 95 °C and one minute at 60 °C. The expression levels of the following genes were determined using TaqMan Gene Expression Assays (all from Applied Biosystems, Foster City, CA, USA): MCP-1, Hs00234140_m1; IL-12a, Hs01073447; TNF-α, Hs99999043_m1; IL-10, Hs00961622_m1; TGF-β3, Hs01086000_m1; TGM-2, Hs00190278_m1, and HPRT1, Hs9999902_m1. qPCR was performed in paired reactions per sample which were normalized to the housekeeping gene HPRT1, serving as internal control. After determining C_t_-values, the 2^−∆∆Ct^ method was used to calculate the n-fold expression of target genes compared to the untreated control.

### 2.6. Immunomediator Expression in hPDLSCs

Primary hPDLSCs were seeded in 6-well plates using 2.5 × 10^5^ cells per well in 3 mL DMEM supplemented with 10% FCS and 1% P/S. After 24 h, hPDLSCs were stimulated with either 10 ng/mL TNF-α or 5 ng/mL IL-1β or 100 ng/mL IFN-γ in the absence or presence of different 1,25(OH)_2_D_3_ concentrations (1–100 nM). For stimulation, the medium was changed to FBS-free DMEM supplemented with 1% P/S to avoid the influence of FBS on the immunomediator expression. After an additional 48 h, the gene expression levels of IDO-1, PTGS-2, and TSG-6 were verified. Additionally, IDO-1 protein levels were calculated by immunostaining and flow cytometry analysis, whereas IDO-1 enzymatic activity was determined by measuring L-kynurenine concentration. The levels of PGE_2_ and TSG-6 were measured by enzyme-linked immunosorbent assay (ELISA).

To determine IDO-1, PTGS-2, and TSG-6 gene expression levels, TaqMan Gene Expression Cells-to-CT Kit (Applied Biosystems, Foster City, CA, USA) was used to lyse cells, reverse transcribe RNA into cDNA, and to perform qPCR. Reverse transcription was performed on a Primus 96 advanced thermocycler heating samples to 37 °C for one hour and to 95 °C for five minutes. qPCR was performed using QuantStudio 3, initially heating samples to 95 °C for 10 min. This was followed by 50 cycles of heating samples to 95 °C for 15 s and to 60 °C for one minute. qPCR was performed in paired reactions using the following TaqMan Gene Expression assays (all from Applied Biosystems, Foster City, CA, USA): IDO-1, Hs00984148_m1; PTGS-2, Hs00153133_m1; TSG-6, Hs00200180, and GAPDH, Hs99999905_m1. The housekeeping gene GAPDH served as an endogenous reference. After determining the C_t_ values for each sample, the n-fold expression of each target gene was calculated, compared to the unstimulated control using the 2^−∆∆Ct^ method.

IDO-1 expression was measured by intracellular immunostaining followed by flow cytometry analysis, similarly to a previously described procedure [38]. For intracellular IDO-1 staining, an Intracellular Fixation and Permeabilization Buffer Set (eBioscience, Waltham, MA, USA) was used to fix and permeabilize 2.5 × 10^5^ hPDLSCs. Intracellular IDO-1 was stained using phycoerythrin (PE)-conjugated mouse anti-human IDO-1 antibody (clone eyedio, eBioscience, Waltham, MA, USA) for 30 min. Unlabeled hPDLSCs and cells stained with PE-conjugated mouse IgG1 K immunoglobulin isotype control (eBioscience, Waltham, MA, USA) functioned as a control. IDO-1 staining was analyzed using FACSCalibur Flow Cytometer. Fluorescence was excited by an argon laser at 488 nm. After analyzing a total of 10,000 cells per sample, the mean fluorescence intensity (m.f.i.) of the IDO-1 positive cells was calculated using the CellQuest 3.3 software (Becton Dickinson, Franklin Lakes, NJ, USA).

IDO-1 enzymatic activities were determined by measuring L-kynurenine concentrations in the conditioned media. Harvested conditioned media were mixed 1:3 (*v*/*v*) with 30% trichloroacetic acid (Sigma-Aldrich, St. Louis, MO, USA) and incubated at 65 °C for 30 min, followed by centrifugation. Next, 125µl supernatant was mixed 1:1 (v/v) with Ehrlich’s Reagent (0.8% P-dimethylbenzaldehyde in glacial acetic acid, Sigma-Aldrich, St. Louis, MO, USA), followed by incubation at room temperature for 10 min. Optical density (OD_492 nm)_ was measured in duplicates photometrically. Standards with known L-kynurenine (Sigma-Aldrich, St. Louis, MO, USA) concentrations ranging from 1000 µM to 7.8 µM were measured photometrically to determine unknown L-kynurenine concentrations. The determined L-kynurenine concentrations were normalized to total protein amounts per sample. The normalized L-kynurenine concentration from unstimulated hPDLSCs was subtracted from each sample.

A Pierce BCA Protein Assay Kit (ThermoFischer Scientific, Waltham, MA, USA) was used to determine the total protein amounts in cell lysates per sample, according to the manufacturer’s protocol. Known concentrations of BSA solutions, ranging from 2000 µg/mL to 31.25 µg/mL, served as a standard. Samples and standards were measured photometrically in duplicates at OD_562_.

TSG-6 protein levels were determined in conditioned media using Human TSG-6 ELISA (RayBiotech, Peachtree Corners, GA, USA), having a detection range between 0.2 ng/mL and 50 ng/mL. PGE_2_ levels were measured in conditioned media using Prostaglandin E_2_ Parameter Assay Kit (R&D Systems, Minneapolis, MN, USA) ranging from 30 pg/mL to 2500 pg/mL.

### 2.7. Statistical Analysis

Differences between appropriate groups were statistically evaluated using Friedman test, followed by Wilcoxon test for pairwise comparison. Statistical analysis was performed with SPSS Statistics 24.0 software (IBM, Armonk, NY, USA). Statistically-significant differences were considered to be at *p*-value < 0.05. To show the extent of the 1,25(OH)_2_D_3_ effect on hPDLSCs, all data points in the presence of 1,25(OH)_2_D_3_ were normalized to the appropriate cytokines.

## 3. Results

### 3.1. 1,25(OH)_2_D_3_ Differently Influences hPDLSC Mediated Suppression of CD4^+^ T Lymphocyte Proliferation Depending on the Presence of TNF-α, IL-1β and IFN-γ

**Figure 1:**
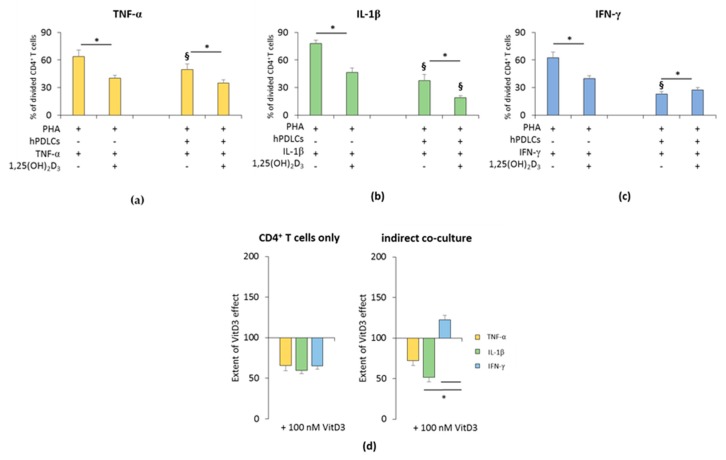
Effect of 1,25(OH)_2_D_3_ on the human periodontal ligament stem cells hPDLSCs mediated suppression of cluster of differentiation (CD)4^+^ T lymphocyte proliferation in the presence of tumor necrosis factor (TNF)-α or interleukin (IL)-1β or interferon (IFN)-γ. Primary hPDLSCs were cocultured with allogenic CD4^+^ T lymphocytes activated by 10 µg/mL phytohemagglutinin (PHA) in the presence of 10 ng/mL TNF-α (**a**) or 5 ng/mL IL-1β (**b**) or 100 ng/mL IFN-γ (**c**) and in the absence or presence of 100 nM 1,25(OH)_2_D_3_. Activated CD4^+^ T lymphocytes treated with different stimuli in the absence of hPDLCs served as the control. After five days, CD4^+^ T lymphocyte proliferation was assessed by carboxyfluorescein succinimidyl ester (CFSE) proliferation assay calculating the percentage of at least once divided CD4^+^ T lymphocytes. (**d**) shows the extent of the 1,25(OH)_2_D_3_ effect on CD4^+^ T lymphocyte proliferation regarding differently-treated hPDLSCs (expressed in % of corresponding cytokine treatment). All data are presented as mean ± S.E.M from five independent experiments using hPDLSCs from five different patients. * Significantly different (*p* < 0.05) compared between groups as indicated. § Significantly lower (*p* < 0.05) compared to appropriate groups without hPDLSCs.

The influence of 1,25(OH)_2_D_3_ on the hPDLSC-mediated suppression of CD4^+^ T lymphocyte proliferation in the presence of TNF-α, IL-1β, or IFN-γ is shown in Figure 1 and Figure S2. All freshly-isolated T lymphocyte populations were CD4 positive (≥93.5% ± 1.10) (Figure S1a). In the absence of hPDLSCs, 1,25(OH)_2_D_3_ significantly inhibited PHA induced CD4^+^ T lymphocyte proliferation in the presence of TNF-α or IL-1β or IFN-γ (Figure 1a–c and Figure S2). The inhibition extent of 1,25(OH)_2_D_3_ was comparable in the presence of TNF-α, IL-1β and IFN-γ (Figure 1d).

In the presence of hPDLSCs treated with TNF-α (Figure 1a and Figure S2a) or IL-1β (Figure 1b and Figure S2b), 1,25(OH)_2_D_3_ significantly strengthened the suppression of CD4^+^ T lymphocyte proliferation. The degree of CD4^+^ T cell proliferation suppression by 1,25(OH)_2_D_3_ was higher in the presence of IL-1β treated hPDLSCs compared to TNF-α, albeit without any significance. In the presence of IFN-γ, the addition of 1,25(OH)_2_D_3_ significantly attenuated hPDLSC induced suppression of CD4^+^ T lymphocyte proliferation (Figure 1c and Figure S2c). The extent of the effect of 1,25(OH)_2_D_3_ in coculture with IFN-γ treated hPDLSCs was significantly different compared to IL-1β and TNF-α. These results indicate that 1,25(OH)_2_D_3_ differently affects CD4^+^ T lymphocyte proliferation in a qualitative and quantitative manner, only in the presence of hPDLSCs treated with different cytokines.

### 3.2. Expression of Pro- and Anti-Inflammatory Cytokines in CD68^+^ Macrophages is Differently Affected by hPDLSCs Primed with 1,25(OH)_2_D_3_ in the Presence of TNF-α or IL-1β or IFN-γ

**Figure 2:**
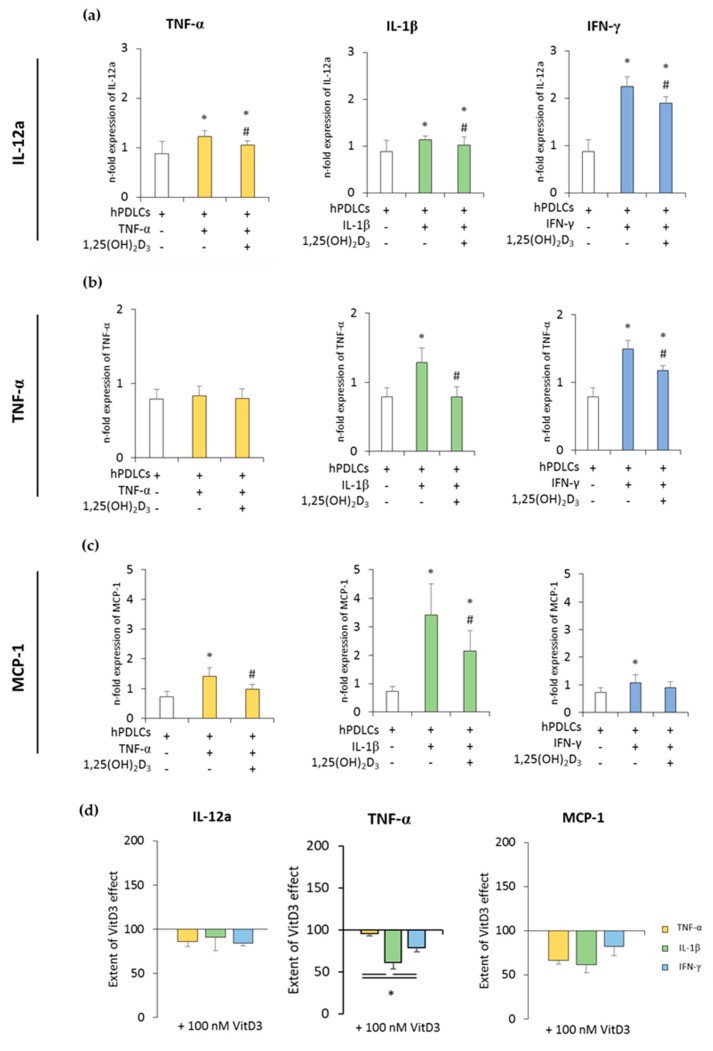
The expression of pro-inflammatory genes in in vitro differentiated CD68^+^ macropahges after coculture with hPDLSCs primed with 1,25(OH)_2_D_3_ and different cytokines. Primary hPDLSCs were primed with 10 ng/mL TNF-α or 5 ng/mL IL-1β or 100 ng/mL IFN-γ in the absence or presence of 100 nM 1,25(OH)_2_D_3_. In vitro differentiated CD68^+^ macrophages were applied to the indirect coculture system with primed hPDLSCs. Gene expression levels of IL-12a (**a**), TNF-α (**b**), and monocyte chemoattractant protein (MCP)-1 (**c**) were determined in macrophages using quantitative polymerase chain reaction (qPCR) after 24 h of coculture. (**a**–**c**) shows the n-fold expression of indicated pro-inflammatory cytokines. In vitro differentiated CD68^+^ macrophages without hPDLSCs served as control (n-fold expression = 1). (**d**) Shows the extent of the effect of 1,25(OH)_2_D_3_ on the macrophage functional status regarding the presence of differently-primed hPDLSCs (expressed in % of corresponding cytokine treatment). All data are presented as mean ± S.E.M. from five independent experiments with hPDLSCs isolated from five different patients. (**a**–**c**): * significantly different (*p* < 0.05) compared to macrophages in the presence of unprimed hPDLSCs. # Significantly different (*p* < 0.05) compared between macrophages in the presence of primed hPDLSCs with and without 1,25(OH)_2_D_3_. (**d**): * significantly different (*p* < 0.05) compared between groups as indicated.

**Figure 3:**
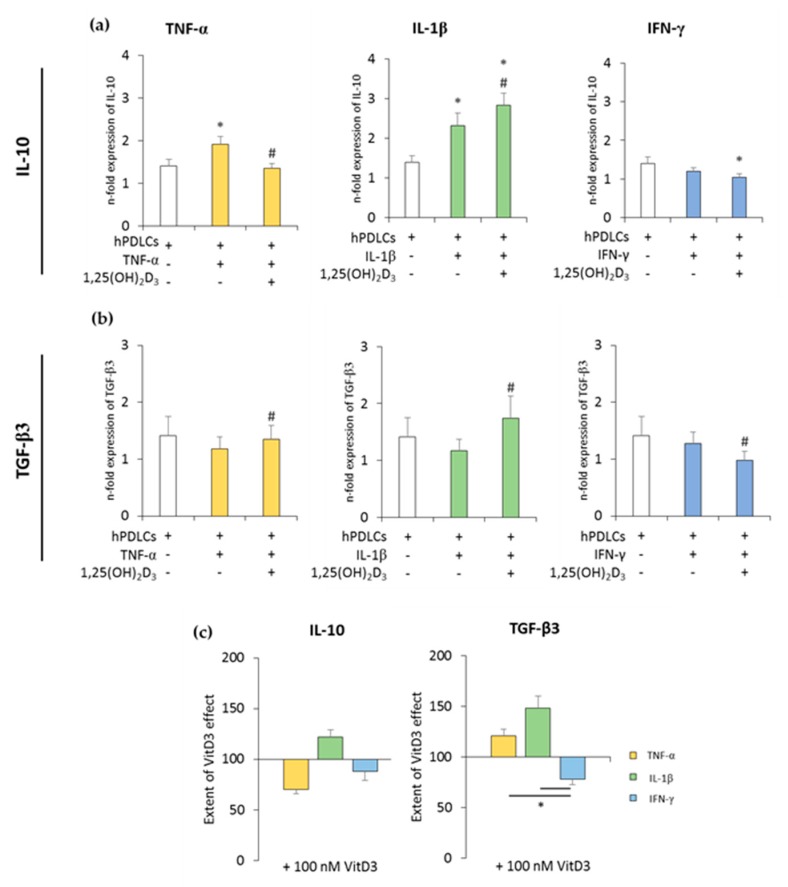
The expression of anti-inflammatory genes in in vitro-differentiated CD68^+^ macropahges after coculture with hPDLSCs primed with 1,25(OH)_2_D_3_ and different cytokines. Primary hPDLSCs were primed with 10 ng/mL TNF-α or 5 ng/mL IL-1β or 100 ng/mL IFN-γ in the absence or presence of 100 nM 1,25(OH)_2_D_3_. In vitro-differentiated CD68^+^ macropahges were applied to the indirect coculture system with primed hPDLSCs. Gene expression levels of IL-10 (**a**) and transforming growth factor (TGF)-β3 (**b**) were verified in macrophages by qPCR after 24 h of coculture. (**a**,**b**) present the n-fold expression of investigated cytokines. Macrophages in the absence of hPDLSCs served as control (n-fold expression = 1). (**c**) shows the extent of the effect of 1,25(OH)_2_D_3_ on macrophage functional status depending on the presence of differently-primed hPDLSCs (expressed in % of corresponding cytokine treatment). All data are presented as mean ± S.E.M, and were received from five independent experiments with hPDLSCs from five different patients. (**a**,**b**): * significantly different (*p* < 0.05) compared to macrophages in the presence of unprimed hPDLSCs. # significantly different (*p* < 0.05) compared between macrophages in the presence of primed hPDLSCs with and without 1,25(OH)_2_D_3_. (**c**): * significantly different (*p* < 0.05) compared between groups as indicated.

Figure 2 and Figure 3 show the expression of pro- and anti-inflammatory cytokines in CD68^+^ macrophages after coculture with hPDLSCs primed with 1,25(OH)_2_D_3_ in the presence of TNF-α, IL-1β, or IFN-γ. All in vitro-differentiated macrophages were positive for CD68 (≥97.1% ± 0.90) (Figure S1b). Unprimed hPDLSCs slightly inhibited the expression of pro-inflammatory cytokines in CD68^+^ macrophages. TNF-α, IL-1β, or IFN-γ priming of hPDLSCs significantly increased expression of all the investigated pro-inflammatory parameters to a variable extent, except for TNF-α expression in coculture with TNF-α-primed hPDLSCs. The presence of 1,25(OH)_2_D_3_ during hPDLSC priming attenuated this enhancement of pro-inflammatory cytokine expression in macrophages. Some quantitative differences in the inhibitory effects of 1,25(OH)_2_D_3_ were observed depending on the priming cytokines. Particularly, TNF-α expression in macrophages induced by the coculture with IL-1β-primed hPDLSCs was inhibited by 1,25(OH)_2_D_3_ more effectively compared to IFN-γ- and TNF-α-primed hPDLSCs (Figure 2d).

The expression of anti-inflammatory cytokines was slightly increased in the coculture with unprimed hPDLSCs. TNF-α- or IL-1β-primed hPDLSCs further significantly increased IL-10 expression to different extents, whereas IFN-γ slightly decreased IL-10 expression. TGF-β3 expression levels were slightly decreased in the presence of cytokine-primed hPDLSCs, albeit without any significance. The presence of 1,25(OH)_2_D_3_ during hPDLSC priming caused different effects on IL-10 and TGF-β3 expression levels. Both qualitative and quantitative differences were observed, depending on the priming cytokines. The presence of 1,25(OH)_2_D_3_ during priming with IL-1β resulted in a significant increase in IL-10 expression in cocultured macrophages. In contrast, the presence of 1,25(OH)_2_D_3_ during priming with TNF-α and IFN-γ led to lower IL-10 expression in macrophages. The presence of 1,25(OH)_2_D_3_ during priming resulted in a significant increase in TGF-β3 expression in macrophages in the case of IL-1β- and TNF-α-primed hPDLSCs, and in a significant decrease in the case of IFN-γ-primed hPDLSCs. Furthermore, significant differences in the effects of 1,25(OH)_2_D_3_ were observed between hPDLSCs primed with different cytokines (Figure 3c). These results indicated that 1,25(OH)_2_D_3_ variably influences CD68^+^ macrophage functional status, depending on the distinctly-primed hPDLSCs in coculture.

### 3.3. 1,25(OH)_2_D_3_ Differently Influences IDO-1 Expression and Enzymatic Activity in hPDLSCs Depending on the Presence of TNF-α or IL-1β or IFN-γ

**Figure 4:**
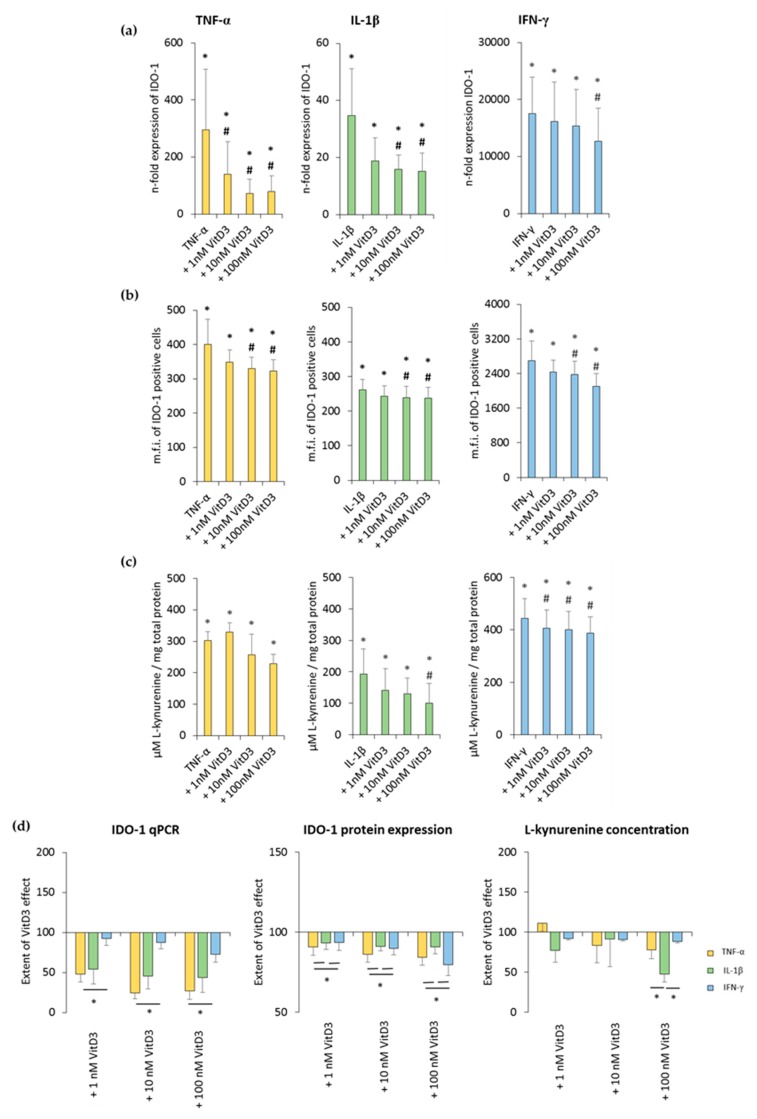
Effect of 1,25(OH)_2_D_3_ on IL-1β or TNF-α or IFN-γ induced indolemaine-2,3-dioxygenase-1 (IDO-1) expression in hPDLSCs. Primary hPDLSCs were treated with 10 ng/mL TNF-α or 5 ng/mL IL-1β or 100 ng/mL IFN-γ in the absence or presence of 1,25(OH)_2_D_3_ (0.01–100 nM). IDO-1 gene expression was analyzed by calculating the n-fold expression compared to unstimulated cells (n-fold expression = 1) using qPCR. GAPDH served as endogenous reference. qPCR data were received from six independent experiments with hPDLSCs from six different individuals (**a**). Intracellular IDO-1 staining and flow cytometry analysis were performed to determine m.f.i. of IDO-1 positive cells. Data were received from five independent experiments with hPDLSCs from five different individuals (**b**). IDO-1 enzymatic activity was verified by measuring L-kynurenine concentrations photometrically in conditioned media. L-kynurenine concentrations were normalized to total protein amounts followed by subtraction of the L-kynurenine concentrations from control group from each sample. Data were received from five independent experiments with hPDLSCs isolated from five different individuals (**c**). (**d**) shows the extent of the 1,25(OH)_2_D_3_ effect regarding differently-induced IDO-1 production in hPDLSCs (expressed in % of corresponding cytokine treatment). All data are presented as mean ± S.E.M. (**a–c**): * significantly higher (*p* < 0.05) compared to unstimulated hPDLSCs. # significantly lower (*p* < 0.05) compared to appropriate cytokine treated hPDLSCs treated in the absence of 1,25(OH)_2_D_3_. (**d**): * significantly different (*p* < 0.05) compared between groups as indicated.

The effects of different 1,25(OH)_2_D_3_ concentrations on TNF-α-, IL-1β-, or IFN-γ-triggered IDO-1 expression and enzymatic activity in hPDLSCs are shown in Figure 4 and Figure S3. All investigated cytokines caused significantly higher IDO-1 gene expression levels to different extents. It was shown that 1,25(OH)_2_D_3_ reduced TNF-α-, IL-1β-, or IFN-γ-induced IDO-1 gene expression levels in a concentration-dependent manner (Figure 4a and Figure S3a). The extent of these suppressions varied, with the highest inhibition being detected for TNF-α-induced IDO-1 gene expression, followed by IL-1β and IFN-γ. The inhibition of IDO-1 gene expression was significantly stronger in the presence of TNF-α compared to IFN-γ (Figure 4d).

Treatment with 1,25(OH)_2_D_3_ caused a concentration-dependent decrease in TNF-α-, IL-1β-, or IFN-γ-induced IDO-1 protein expression. A significant decrease was observed starting at 10 nM 1,25(OH)_2_D_3_ in the presence of all three cytokines (Figure 4b and Figure S3b). It was shown that 1,25(OH)_2_D_3_ caused a stronger decrease in IDO-1 protein expression in the presence of TNF-α and IFN-γ (Figure 4d).

Treatment with all three cytokines caused a significant increase in L-kynurenine concentration in conditioned media. It was shown that 1,25(OH)_2_D_3_ decreased L-kynurenine concentrations in a concentration-dependent manner (Figure 4c and Figure S3c). Depending on the cytokines, 1,25(OH)_2_D_3_ suppressed L-kynurenine production to different extents. Notably, 100 nM 1,25(OH)_2_D_3_ showed a significantly stronger inhibition with IL-1β compared to TNF-α and IFN-γ (Figure 4d). These results indicate that 1,25(OH)_2_D_3_ differently influences IDO-1 expression and its enzymatic activity in hPDLSCs, depending on cytokine treatment.

### 3.4. 1,25(OH)_2_D_3_ Differently Influences TSG-6 Expression in hPDLSCs, Depending on the Presence of TNF-α or IL-1β or IFN-γ

**Figure 5:**
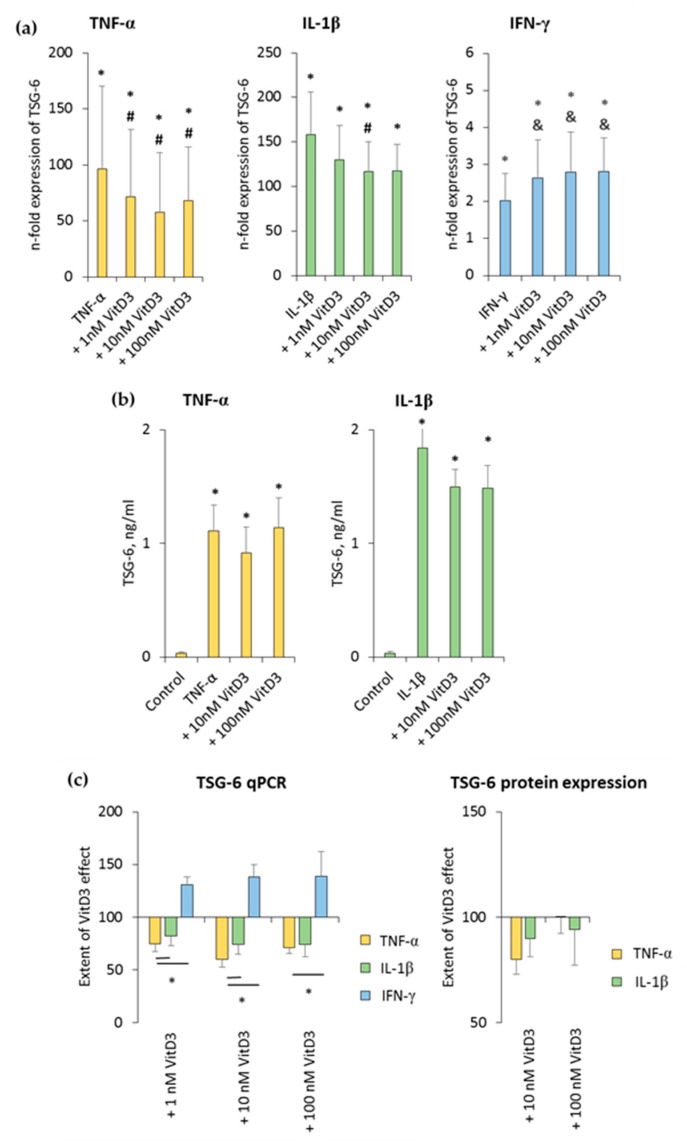
Effect of 1,25(OH)_2_D_3_ on IL-1β or TNF-α or IFN-γ induced tumor necrosis factor inducible gene (TSG)-6 expression in hPDLSCs. Primary hPDLSCs were stimulated with either 10 ng/mL TNF-α or 5 ng/mL IL-1β or 100 ng/mL IFN-γ in the absence or presence of 1,25(OH)_2_D_3_ (0.01–100 nM). TSG-6 gene expression (**a**) was analyzed by determining n-fold expression levels compared to unstimulated hPDLSCs (n-fold expression = 1) using qPCR. GAPDH served as endogenous control. (**b**) shows TSG-6 protein levels in conditioned media determined by enzyme-linked immunosorbent assay (ELISA). Notably, the levels of TSG-6 were below the detection limit after IFN-γ stimulation, and therefore, are not shown. (**c**) shows the extent of the 1,25(OH)_2_D_3_ effect on differently-induced TSG-6 expression in hPDLSCs (expressed in % of corresponding cytokine treatment). All data are presented as mean ± S.E.M. and were received from five independent experiments using hPDLSCs from five different patients. (**a**,**b**): * significantly higher (*p* < 0.05) compared to unstimulated hPDLSCs. # significantly lower (*p* < 0.05) compared to appropriate cytokine treated hPDLSCs in the absence of 1,25(OH)_2_D_3_. & significantly higher (*p* < 0.05) compared to appropriate cytokine treated hPDLSCs in the absence of 1,25(OH)_2_D_3_. (**c**): * significantly different (*p* < 0.05) compared between groups as indicated.

The effect of 1,25(OH)_2_D_3_ on TNF-α-, IL-1β-, or IFN-γ-induced TSG-6 expression in hPDLSCs is shown in Figure 5 and Figure S4. TSG-6 gene expression was significantly enhanced by all inflammatory stimuli to different extents. TNF-α- and IL-1β-induced TSG-6 gene expression was inhibited by 1,25(OH)_2_D_3_, whereas IFN-γ-induced TSG-6 expression was increased by 1,25(OH)_2_D_3_ in a concentration-dependent manner (Figure 5a and Figure S4a). The extent of 1nM and 100 nM 1,25(OH)_2_D_3_ suppression effects was significantly stronger in the presence of TNF-α compared to IL-1β. Additionally, the extent of 1,25(OH)_2_D_3_ effect significantly differed between TNF-α- and IFN-γ-treated hPDLSCs (Figure 5c).

TNF-α and IL-1β significantly induced TSG-6 protein levels to slightly different extents. It was shown that 10 nM 1,25(OH)_2_D_3_ slightly reduced TNF-α-induced TSG-6 protein levels, whereas in the presence of IL-1β, TSG-6 protein levels were reduced by 10 nM and 100 nM 1,25(OH)_2_D_3_ (Figure 5b and Figure S4b). The extent of 1,25(OH)_2_D_3_ suppression slightly changed, depending on the cytokine and 1,25(OH)_2_D_3_ concentration, albeit without any significance. In the presence of TNF-α, 10 nM 1,25(OH)_2_D_3_ showed a stronger TSG-6 protein expression suppression, whereas in the presence of IL-1β, 100 nM 1,25(OH)_2_D_3_ caused a stronger suppression of TSG-6 protein expression (Figure 5c). IFN-γ caused TSG-6 protein levels which were out of the ELISA detection range. Consequently, changes in TSG-6 protein levels were not detectable in the presence of IFN-γ. These results indicate that TSG-6 expression in hPDLSCs is differently affected by 1,25(OH)_2_D_3_, depending on the presence of different stimuli, which even cause opposite effects of 1,25(OH)_2_D_3_.

### 3.5. 1,25(OH)_2_D_3_ Differently Influences PTGS-2 Expression in hPDLSCs, Depending on the Presence of TNF-α or IL-1β or IFN-γ

**Figure 6:**
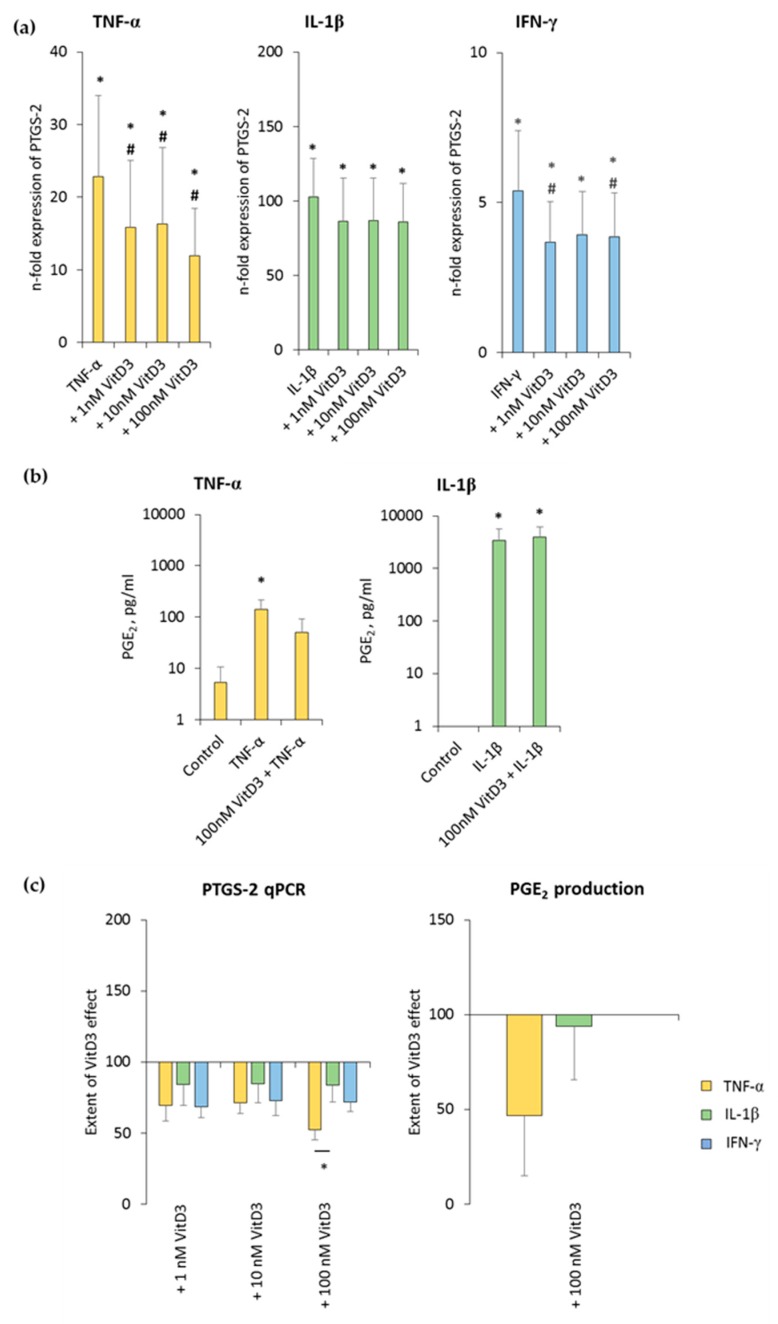
Effect of 1,25(OH)_2_D_3_ on IL-1β or TNF-α or IFN-γ induced prostaglandin-endoperoxide synthase (PTGS)-2/prostaglandin E_2_ (PGE_2_) production in hPDLSCs. Primary hPDLSCs were stimulated with either 10 ng/mL TNF-α or 5 ng/mL IL-1β or 100 ng/mL IFN-γ in the absence or presence of 1,25(OH)_2_D_3_ (0.01–100 nM). PTGS-2 gene expression (**a**) was analyzed by determining n-fold expression levels compared to unstimulated hPDLSCs (n-fold expression = 1) using qPCR. GAPDH functioned as an endogenous reference. (**b**) shows PGE_2_ levels in conditioned media determined by ELISA. Notably, the levels of PGE_2_ were below the lower detection limit after IFN-γ stimulation and therefore are not shown. (**c**) shows the extent of the 1,25(OH)_2_D_3_ effect on differently-induced PTGS-2 gene expression and on differently-induced PGE_2_ production in hPDLSCs (expressed in % of corresponding cytokine treatment). All data are presented as mean ± S.E.M. and were received from five independent experiments using hPDLSCs from five different patients. (**a**): * significantly higher (*p* < 0.05) compared to unstimulated hPDLSCs. # significantly lower (*p* < 0.05) compared to appropriate cytokine treated hPDLSCs in the absence of 1,25(OH)_2_D_3_. (**b**): * significantly different (*p* < 0.05) compared between groups as indicated. (**c**): * significantly different (*p* < 0.05) compared between groups as indicated.

The effect of various 1,25(OH)_2_D_3_ concentrations on differently-stimulated PTGS-2 and PGE_2_ production in hPDLSCs is shown in Figure 6 and Figure S5. All three inflammatory stimuli caused a significant enhancement of PTGS-2 gene expression, albeit by different extents. 1,25(OH)_2_D_3_ reduced PTGS-2 gene expression in a concentration-dependent manner, independently from used cytokine type (Figure 6a and Figure S5a). It was found that 100 nM 1,25(OH)_2_D_3_ showed slightly different inhibition extents, causing a significantly stronger suppression in the presence of TNF-α, followed by IFN-γ and IL-1β (Figure 6c).

TNF-α and IL-1β significantly induced the PGE_2_ levels to a clearly different extent, and 100 nM 1,25(OH)_2_D_3_ slightly reduced TNF-α-induced PGE_2_ production, without any significance. In the presence of IL-1β, 1,25(OH)_2_D_3_ did not affect PGE_2_ production (Figure 6b and Figure S5b). The extent of 1,25(OH)_2_D_3_ suppression of PGE_2_ production was clearly stronger in the presence of TNF-α compared to IL-1β, albeit without any significance (Figure 6c). IFN-γ caused PGE_2_ levels which were out of the ELISA detection range. Consequently, changes in PGE_2_ levels were not detectable in the presence of IFN-γ. These results indicate that PTGS-2 expression and PGE_2_ production in hPDLSCs are differently affected by 1,25(OH)_2_D_3_.

## 4. Discussion

The role of vitamin D_3_ in bone metabolism [1] and its function in regulating the immune system [2] are well established. Several clinical studies showed an association between vitamin D_3_ deficiency and periodontitis, an inflammatory disorder affecting periodontal tissue, but their results are partially inconclusive [31,32,39]. In vitro studies show an anti-inflammatory effect of vitamin D_3_ in lipopolysaccharide (LPS) -induced response in hPDLSCs [7,33,34,35]. However, an inflammatory response in periodontitis is driven by various cytokines, which might be expressed to varying degrees depending on disease stage and type. Several in vitro studies have already reported different immunomodulatory activities of hPDLSCs, depending on the presence of various cytokines [21,22,23]. However, the combined immunomodulatory effect of vitamin D_3_ and hPDLSCs treated with various cytokines has never been investigated. Hence, this in vitro study, for the first time, directly compared the effect of 1,25(OH)_2_D_3_ in the presence of TNF-α, IL-1β, or IFN-γ on the immunomodulatory activities of hPDLSCs using indirect coculture models with either CD4^+^ T lymphocytes or CD68^+^ macrophages. Indirect coculture models allow paracrine to interact between different cell types and make it possible to easily separate them for subsequent analysis. However, such models have also some limitations, because they do not consider direct cell-to-cell interactions, which also mediate some immunomodulatory effects. Further, we focused on purified CD4^+^ T lymphocytes and macrophages to minimize indirect effects of hPDLSCs on these cells via other immune cells which are enclosed in PBMCs. Additionally, the combined effect of 1,25(OH)_2_D_3_ with TNF-α, IL-1β, or IFN-γ on the expression of immunomediators in hPDLSCs was investigated. The main observation of this study is that the immunomodulatory effect of 1,25(OH)_2_D_3_ via hPDLSCs depends on the local cytokine milieu. It was shown that 1,25(OH)_2_D_3_ differently affected hPDLSC immunomediator expression and their immunomodulatory activities toward CD4^+^ T lymphocytes and CD68^+^ macrophages.

Our data showed that the effect of 1,25(OH)_2_D_3_ on PHA-stimulated CD4^+^ T lymphocyte proliferation is drastically modified by hPDLSCs, and that it strongly depends on the environment. In monoculture, the extent of the inhibitory effect of 1,25(OH)_2_D_3_ on CD4^+^ T lymphocyte proliferation was not influenced by different cytokines. In the presence of hPDLSCs, the proliferation of CD4^+^ T lymphocytes was inhibited to different extents, depending on the type of cytokine present. Furthermore, under these conditions, the effect of 1,25(OH)_2_D_3_ on CD4^+^ T lymphocyte proliferation depended on TNF-α, IL-1β, and IFN-γ. Notably, in the presence of IFN-γ, hPDLSCs converted the suppression effect of 1,25(OH)_2_D_3_ into a stimulating effect on CD4^+^ T lymphocyte proliferation, which was in contrast with TNF-α and IL-1β. This indicates that the variable effects of 1,25(OH)_2_D_3_ are mainly mediated by hPDLSCs. In our study, PHA was used for CD4^+^ T lymphocyte stimulation, which is a widely-accepted approach [40]. The validity of the obtained results should be confirmed for other T lymphocyte activation methods, like anti-CD3/anti-CD28 antibodies or concanavalin A. Another limitation of CD4^+^ T lymphocyte proliferation measurements was that we did not detect the percentage of apoptotic cells. As shown by previous studies, in vitro T lymphocyte proliferation is accompanied by apoptosis [41]. Although, one previous study showed that hPDLSCs have no significant effect on T lymphocyte apoptosis in coculture [21], this might still influence the interpretation of our data.

As the next step, we investigated the effect of hPDLSCs primed with inflammatory cytokines and 1,25(OH)_2_D_3_ on the expression of different proteins in macrophages. We determined the expression of pro-inflammatory TNF-α, IL-12a, and MCP-1, as well as anti-inflammatory IL-10 and TGF-β3 genes. Verifying the expression of the investigated cytokines on protein levels was not possible in this coculture model, because most investigated cytokines are produced by both macrophages and hPDLSCs [42], which makes it impossible to identify their origin. In this part, we used primed cells to avoid the direct effect of different substances on the macrophages. Our results showed that priming of hPDLSCs with 1,25(OH)_2_D_3_ together with different cytokines strongly influenced the gene expression in primary macrophages. Moreover, the presence of 1,25(OH)_2_D_3_ during hPDLSC priming caused an alteration in the gene expression in macrophages, depending on the type of cytokine. The effect of 1,25(OH)_2_D_3_ was strongly anti-inflammatory, i.e., associated with lower expression of pro-inflammatory and higher expression of anti-inflammatory genes, which was only observed in the case of IL-1β primed hPDLSCs. The application of 1,25(OH)_2_D_3_ together with TNF-α or IFN-γ resulted in diminishing of the expression of both pro- and anti-inflammatory genes in cocultured macrophages, and thus, did not have clear anti-inflammatory effects. Hence, similar to CD4^+^ T lymphocytes, the hPDLSC-mediated effect on macrophages is not strongly anti-inflammatory, and depends on the microenvironment.

Modifying the immunomodulatory activities of hPDLSCs toward CD4^+^ T lymphocyte and macrophages by 1,25(OH)_2_D_3_ and its dependency on the inflammatory environment could be associated with the alteration in the expression of immunomodulatory proteins in hPDLSCs. In our study we determined the expression of three major immunomediators, namely IDO-1, PTGS-2, PGE_2_, and TSG-6. IDO-1 and PGE_2_ are known to mediate the inhibitory effect of hPDLSCs on T lymphocytes. The immunomodulatory effects of hPDLSCs on macrophages are among others mediated by PGE_2_ and TSG-6. In most cases, our study demonstrated the inhibitory effect of 1,25(OH)_2_D_3_ on the expression of immunomodulatory factors in hPDLSCs [18]. On the one hand, we showed that decreased expression of these mediators in hPDLSCs by 1,25(OH)_2_D_3_ should result in pro-inflammatory effects. On the other hand, previous studies show that 1,25(OH)_2_D_3_ decreases expression of pro-inflammatory cytokines in hPDLSCs, which implies anti-inflammatory effects [7,33,34,35]. Thus, it seems that 1,25(OH)_2_D_3_ causes both anti- and pro-inflammatory immunomodulatory activities in hPDLSCs, and the balance between these effects depends on the microenvironment. TNF-α, IL-1β, and IFN-γ activate specific signaling pathways, resulting in specific immunomediator expression profiles, and 1,25(OH)_2_D_3_ may differently affect these signaling pathways [21,22,23].

We investigated the effect of 1,25(OH)_2_D_3_ in the presence of various cytokines. All used cytokines are assumed to play an important role in different phases of inflammatory responses. Particularly, TNF-α and IL-1β are produced in large amounts by polymorphonuclear leukocytes and activated macrophages during the initial stages of infection. IFN-γ is a specific cytokine produced during Th1 response, which is involved in progressive periodontal lesions. The concentration of IL-1β and IFN-γ used in our experiments were similar to those observed in the gingival crevicular fluid (GCF) of periodontitis patients [43,44]. In contrast, the concentration of TNF-α used in our study was markedly higher compared to those measured in GCF in periodontitis (up to 100 pg/mL) [45]. However, this concentration does not entirely reflect those in the intercellular space, which may be higher than that in GCF under certain circumstances, for example in a proximity to active immune cells. Therefore, we assume that the described effect might be physiologically relevant during different the phases of inflammatory response.

By translating our results into an in vivo situation, we can conclude that 1,25(OH)_2_D_3_ fine-tunes periodontal tissue homeostasis partly through hPDLSCs. In periodontal disease, as in many inflammatory disorders, immune response plays a dual role, eliminating periodontal pathogens on the one hand, but also causing collateral tissue damage on the other [46]. The balance between the activation and resolution of periodontal inflammation is regulated by complex mechanisms including several cell types [47]. Vitamin D_3_ may affect this balance between the activation and resolution of the inflammatory response in periodontitis, and thus, contribute to the disease progression. It seems that the effects of 1,25(OH)_2_D_3_ are very complex and strongly depend on environmental factors. This should be considered by vitamin D_3_ supplementation in inflammatory diseases, and particularly during initial periodontal therapy. It should be also noted that the role of vitamin D_3_ in periodontal tissue homeostasis is not limited to its effect on immune cells and the resident tissue-specific MSC-like cells. Previous studies have shown that vitamin D_3_ induces the production of antimicrobial peptides, and that this will definitely influence host-microbial homeostasis in the oral cavity [48].

## 5. Conclusions

In conclusion, this in vitro study indicates that 1,25(OH)_2_D_3_ differently influences the immunomodulatory effects of hPDLSCs toward CD4^+^ T lymphocytes and CD68^+^ macrophages, depending qualitatively and quantitatively on inflammatory cytokines. These variable effects of 1,25(OH)_2_D_3_ may differently fine-tune the local immune response in the periodontium, and consequently, the balance between periodontal pathogen elimination and periodontal tissue destruction during periodontitis. Importantly, this seems to depend on the local microenvironment. Further studies are needed to investigate the exact role of 1,25(OH)_2_D_3_ on local inflammatory processes in the periodontal ligament and during periodontitis progression.

## Supplementary Materials

The following are available online at https://www.mdpi.com/2077-0383/8/12/2211/s1, Figure S1: Representative purity of CD4^+^ T lymphocyte and CD68^+^ macrophage populations; Figure S2: Effect of 1,25(OH)_2_D_3_ on the hPDLSCs mediated suppression of CD4^+^ T lymphocyte proliferation in the presence of TNF-α or IL-1β or IFN-γ; Figure S3: Effect of 1,25(OH)_2_D_3_ on IL-1β or TNF-α or IFN-γ induced IDO-1 expression in hPDLSCs in different donors; Figure S4: Effect of 1,25(OH)_2_D_3_ on IL-1β or TNF-α or IFN-γ induced TSG-6 expression in hPDLSCs in different donors; Figure S5: Effect of 1,25(OH)_2_D_3_ on IL-1β or TNF-α or IFN-γ induced PTGS-2/PGE2 production in hPDLSCs in different donors.
