# Supplementary material for: 1,25(OH)2D3 Differently Affects Immunomodulatory Activities of Mesenchymal Stem Cells Depending on the Presence of TNF-α, IL-1β and IFN-γ
Source: J Clin Med. 2019 Dec 14;8(12):2211. doi: 10.3390/jcm8122211 (PMC6947512; doi:10.3390/jcm8122211)
Supplement: Supplementary file 1 [file jcm-08-02211-s001.pdf]

Journal of
Clinical Medicine

MDPI

Supplementary Materials

Original research article

1,25(OH)2D3 Differently Affects Immunomodulatory Activities of Mesenchymal Stem Cells depending on the presence of TNF-α, IL-1β and IFN-γ

Christian Behm [1], Alice Blufstein [2], Johannes Gahn [3], Barbara Kubin [4], Michael Nemec[5], Andreas Moritz[6] Xiaohui Rausch-Fan [7] and Oleh Andrukhov [8*]

Supplementary Figure 1

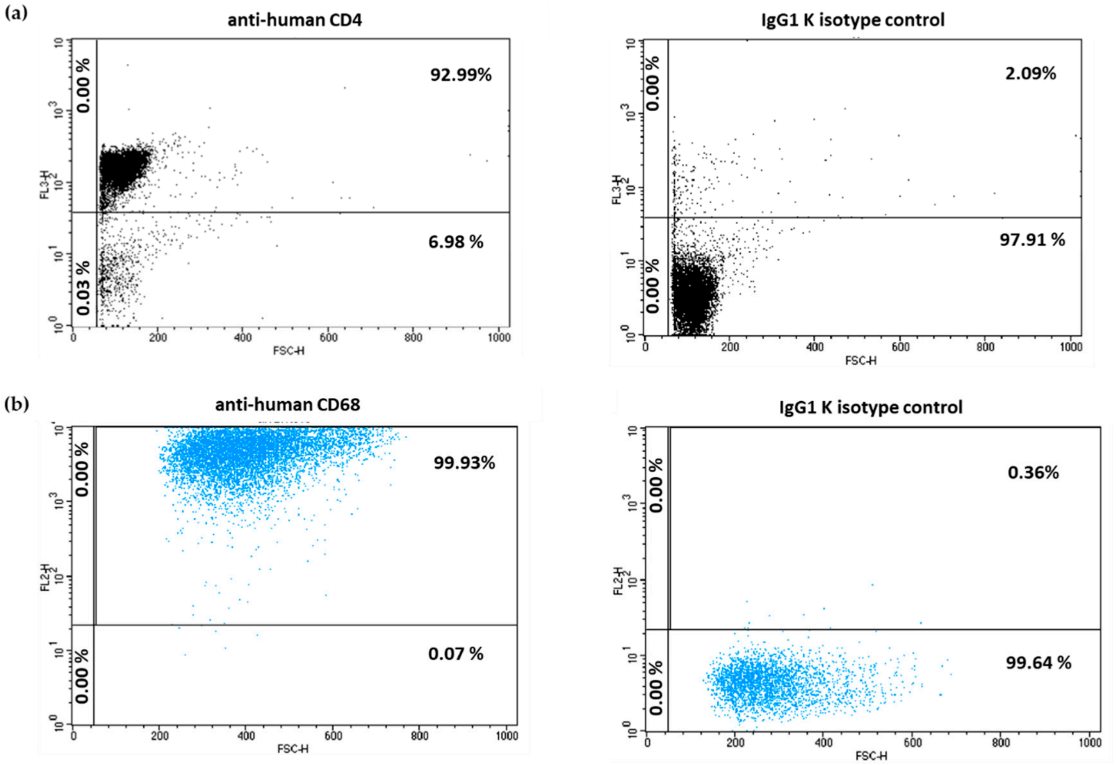

Figure S1. Representative purity of CD4⁺ T lymphocyte and CD68⁺ macrophage populations. (a) shows dot plots with the percentage of CD4 positive T lymphocytes. Initial purity of isolated CD4 positive T lymphocytes was verified by CD4 immunostaining using PerCyp Cyanine 5.5-conjugated mouse anti-human CD4, clone RPA-T4 antibody (Thermo Fischer Scientific, Waltham, USA) and 3% BSA solution supplemented with 0.09% sodium azid. PerCyp Cyanine 5.5-conjugated IgG1 K antibody served as isotype control. (b) shows dot blots with the percentage of CD68 positive macrophages in *in vitro* differentiated macrophage population. Isolated CD14⁺ monocytes were differentiated *in vitro* to macrophages by stimulation with 50 ng/ml M-CSF. Purity of *in vitro* differentiated macrophages was verified by CD68 immunostaining using PE-conjugated mouse

anti-human CD68, clone eBioY1/82A antibody (Thermo Fischer Scientific, Waltham, USA) and 3% BSA solution supplemented with 0.09% sodium azid. PE-conjugated IgG K antibody served as isotype control. CD4 and CD68 immunostaining was analysed by flow cytometry using FACSCalibur Flow Cytometer and CellQuest 3.3 software. Total number of counted cells was limited to 10.000 events.

Supplementary Figure 2

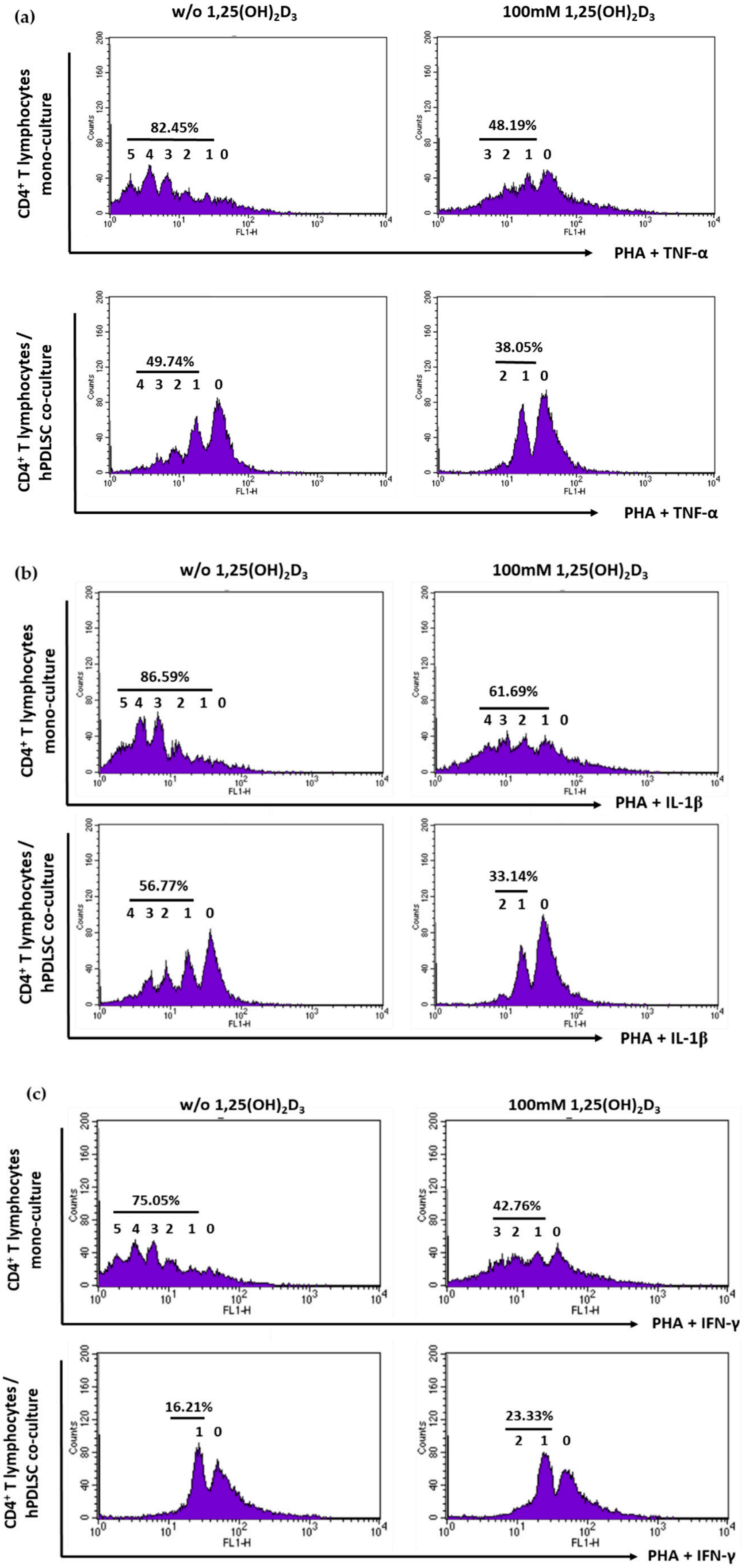

Figure S2. Effect of $1,25(OH)_2D_3$ on the hPDLSCs mediated suppression of $CD4^+$ T lymphocyte proliferation in

the presence of TNF-α or IL-1β or IFN-γ. Representative results of CD4+ T lymphocyte proliferation assay are presented in one-parameter histograms. The percentage of at least once divided CD4+ T lymphocyte are given. The number 0 indicates the CD4+ T lymphocyte parental generation, whereas 1, 2, 3, 4 and 5 shows the first, second, third, fourth and fifth generation, respectively.

Supplementary Figure 3

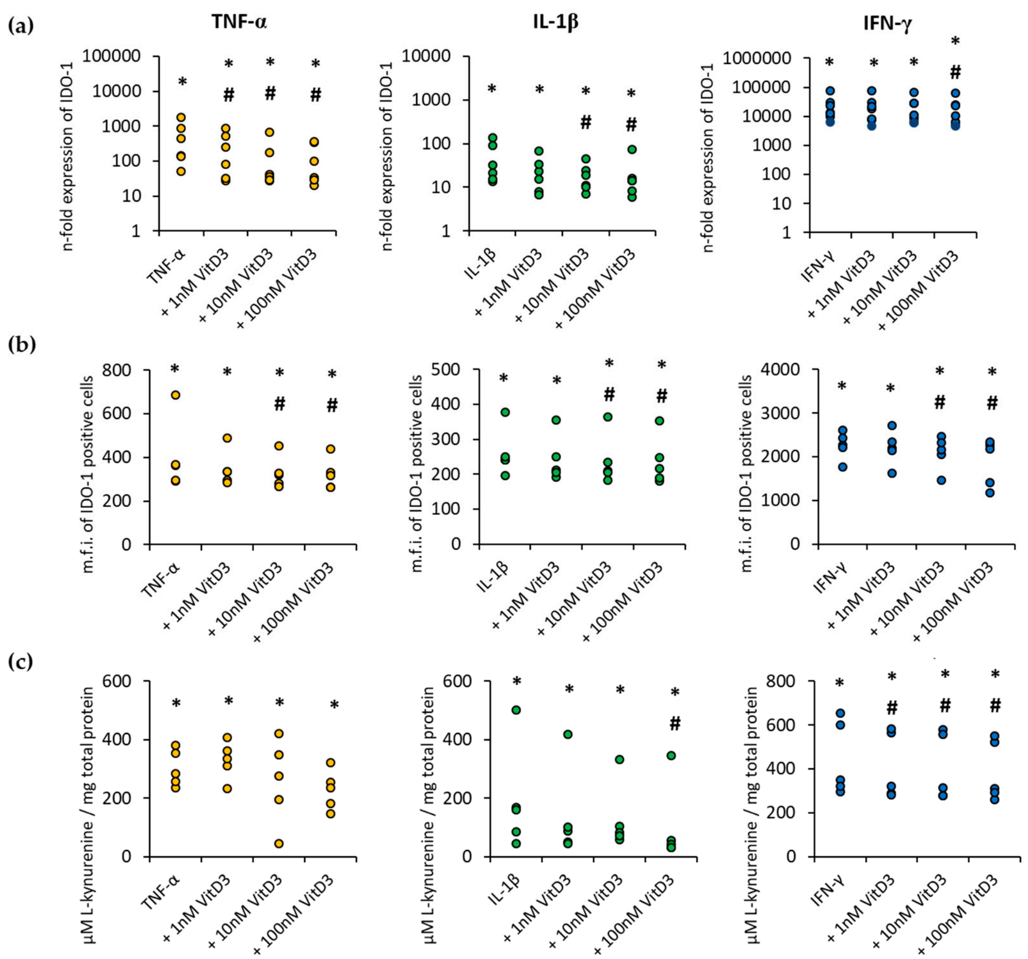

Figure S5. Effect of 1,25(OH)₂D₃ on IL-1β or TNF-α or IFN-γ induced IDO-1 expression in hPDLSCs in different donors. Each single point shows for IDO-1 gene expression levels (a), IDO-1 protein levels (b) and measured L-kynurenine concentrations (c) measured in cells of individual donor included in Figure 4. * significantly higher ($p < 0.05$) compared to unstimulated hPDLSCs. # significantly lower ($p < 0.05$) compared to appropriate cytokine treated hPDLSCs treated in the absence of 1,25(OH)₂D₃.

Supplementary Figure 4

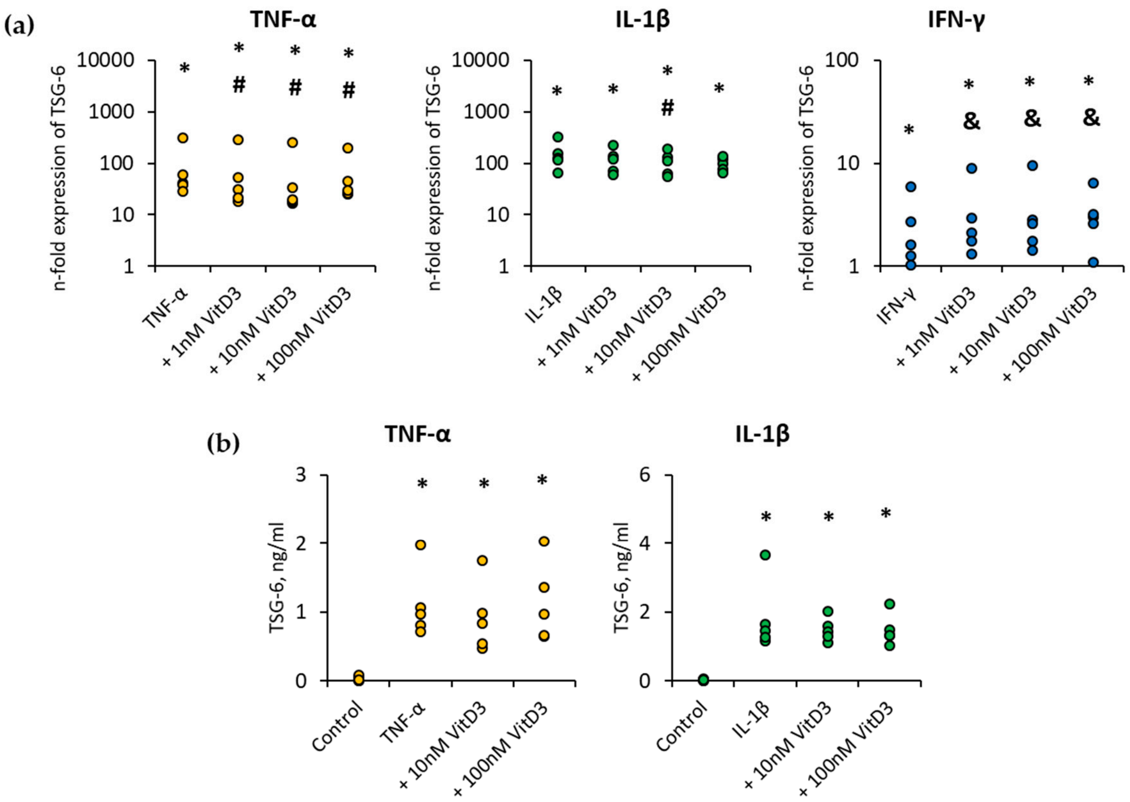

Figure S6. Effect of 1,25(OH)$_2$D$_3$ on IL-1β or TNF-α or IFN-γ induced TSG-6 expression in hPDLSCs in different donors. Each single point shows TSG-6 gene expression levels (a) and TSG-6 protein concentrations (b) measured in cells of individual donors included in Figure 5. * significantly higher ($p < 0.05$) compared to unstimulated hPDLSCs. # significantly lower ($p < 0.05$) compared to appropriate cytokine treated hPDLSCs treated in the absence of 1,25(OH)$_2$D$_3$. & significantly higher ($p < 0.05$) compared to appropriate cytokine treated hPDLSCs in the absence of 1,25(OH)$_2$D$_3$.

Supplementary Figure 5

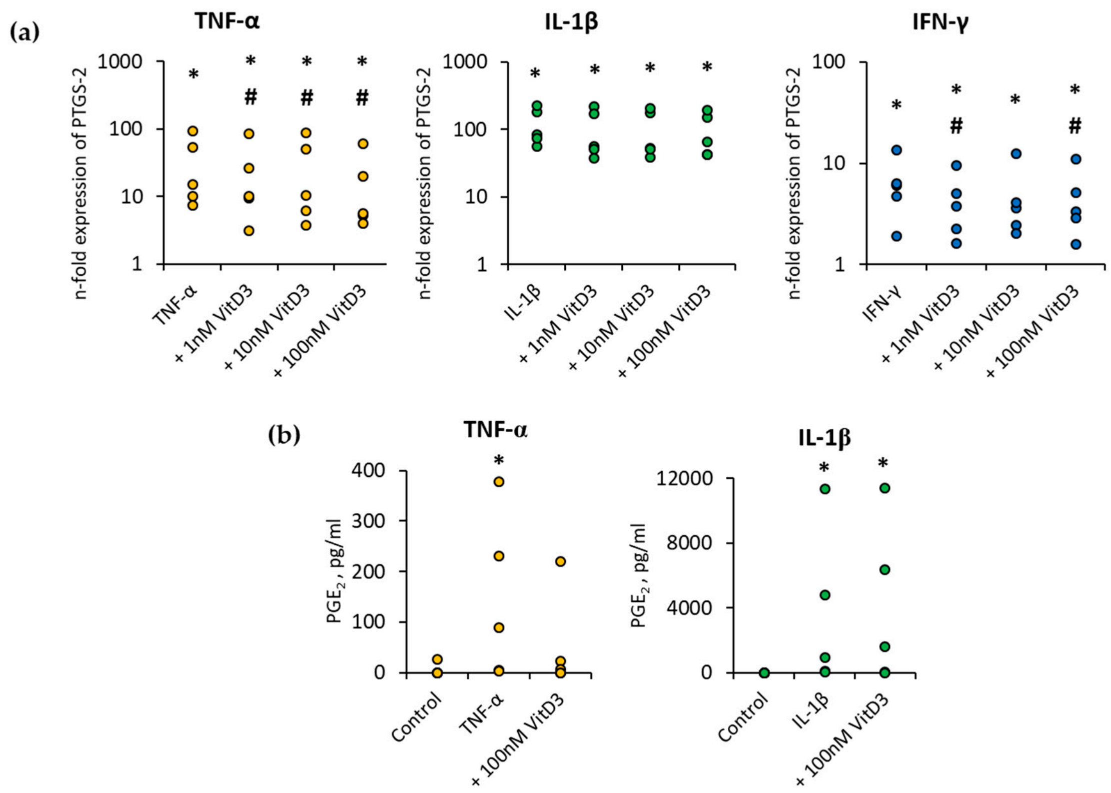

Figure S7. Effect of 1,25(OH)$_2$D$_3$ on IL-1β or TNF-α or IFN-γ induced PTGS-2/PGE$_2$ production in hPDLSCs in different donors. Each single point shows PTGS-2 gene expression (a) and PGE$_2$ concentrations (b) measured in cells of individual donor included in Figure 6. * significantly higher ($p < 0.05$) compared to unstimulated hPDLSCs. # significantly lower ($p < 0.05$) compared to appropriate cytokine treated hPDLSCs in the absence of 1,25(OH)$_2$D$_3$.
